# Parental use of routines, setting limits, and child screen use during COVID-19: findings from a large Canadian cohort study

**DOI:** 10.3389/frcha.2024.1293404

**Published:** 2024-02-21

**Authors:** Amanda Lien, Xuedi Li, Charles D. G. Keown-Stoneman, Katherine T. Cost, Leigh M. Vanderloo, Sarah Carsley, Jonathon Maguire, Catherine S. Birken

**Affiliations:** ^1^ Temerty Faculty of Medicine, University of Toronto, Toronto, ON, Canada; ^2^ Child Health Evaluative Sciences, SickKids Research Institute, Toronto, ON, Canada; ^3^ Dalla Lana School of Public Health, University of Toronto, Toronto, ON, Canada; ^4^ Department of Research & Evaluation, Applied Health Research Centre (AHRC), Li Ka Shing Knowledge Institute, St. Michael’s Hospital, Unity Health Toronto, Toronto, ON, Canada; ^5^ Department of Psychiatry & Behavioural Neurosciences, McMaster University, Hamilton, ON, Canada; ^6^ Research Institute, The Hospital for Sick Children, Toronto, ON, Canada; ^7^ ParticipACTION, Toronto, ON, Canada; ^8^ School of Occupational Therapy, Western University, London, ON, Canada; ^9^ Department of Health Promotion, Chronic Disease and Injury Prevention, Public Health Ontario, Toronto, ON, Canada; ^10^ Department of Paediatrics, Temerty Faculty of Medicine, University of Toronto, Toronto, ON, Canada; ^11^ Department of Nutritional Sciences, Temerty Faculty of Medicine, University of Toronto, Toronto, ON, Canada; ^12^ Department of Pediatrics, St. Michael’s Hospital, Unity Health Toronto, Toronto, ON, Canada; ^13^ Li Ka Shing Knowledge Institute, St. Michael’s Hospital, Unity Health Toronto, Toronto, ON, Canada; ^14^ Division of Paediatric Medicine, The Hospital for Sick Children, Toronto, ON, Canada

**Keywords:** child screen time, COVID-19, limit setting, routines, Canadian 24-hour Movement Guidelines

## Abstract

**Background:**

An increase in child screen time has been observed throughout the COVID-19 pandemic. Home environment and parenting practices have been associated with child screen time. The purpose of this study was to examine associations between parental use of routines, limit setting, and child screen time during the (COVID-19) pandemic to inform harm-reducing strategies to limit the potential harms ensued by excessive screen use.

**Methods:**

A cohort study was conducted in 700 healthy children (3,628 observations) aged 0–11 years though the TARGet Kids! COVID-19 Study of Children and Families in Toronto, Canada from May 2020-May 2021. The independent variables assessed were parent-reported use of routines and setting limits. Outcomes were parent-reported child daily screen time in minutes and whether the Canadian 24-Hour screen time guideline was met, defined as 0 for <1 years, 60 or less for 1–5 years, and 120 or less for >5 years. Linear and logistic mixed-effects models were fitted using repeated measures of independent variables and outcomes with *a priori* stratification by developmental stages (<3, 3–4.99, ≥5 years).

**Results:**

A total of 700 children with 3,628 observations were included in this study [mean age = 5.5 (SD = 2.7, max = 11.9) years, female = 47.6%]. Mean change in child screen time before vs. during the pandemic was +51.1 min/day and level of parental use of routines and setting limits remained stable. Lower use of routines was associated with higher child screen time (*β* = 4.0 min; 95% CI: 0.9, 7.1; *p* = 0.01) in ages ≥5 years and lower odds of meeting the screen time guideline in ages <3 years and ≥5 years (OR = 0.59; 95% CI: 0.38, 0.88; *p* = 0.01; OR = 0.76; 95% CI: 0.67, 0.87; *p *< 0.01). Lower use of limit setting was associated with higher child screen time and lower odds of meeting the screen time guideline in ages ≥5 years (*β* = 3.8 min; 95% CI: 0.69, 6.48; *p *< 0.01; OR = 0.86; 95% CI: 0.78, 0.94; *p *< 0.01).

**Conclusions:**

Lower parental use of routines and limits during the COVID-19 pandemic were associated with higher screen time and lower odds of meeting the screen time guideline among school-age children. Results may help inform strategies to promote healthy screen use in this age group.

## Background

Public health prevention measures have been implemented to combat the transmission of coronavirus disease (COVID-19) (1). During this period, substantial increases in child and adolescent screen time have been observed (2–4). While prosocial and educational content and parental engagement during child screen use can be beneficial, excessive use of all forms of digital media are associated with developmental and health concerns. The American Academy of Pediatrics has therefore suggested that guidance surrounding screen use include resources and realistic strategies that help parents monitor and limit screen time (5). The Canadian Society for Exercise Physiology has also established the Canadian 24-hour Movement Guidelines, which describes recommended amounts of sleep, screen time, and physical activity by age group (6). Meeting these guidelines have been associated with various positive health outcomes, prior to and during the COVID-19 pandemic (3, 7, 8).

Greater recreational screen time has been associated with various negative physical, social, and mental health indicators among school-age children and youth (9–11), both prior and during the COVID-19 pandemic, such as obesity, cardiometabolic factors, emotional problems, and prosocial behaviours. These negative associations also extend to children less than 5 years of age, where poorer sleep outcomes and psychosocial health and increased adiposity, motor or cognitive developmental delay have been observed (12, 13).

An increase in screen time was reported among Canadian children and youth (5–17 years) prior to during the COVID-19 pandemic (3, 14). Similarly, studies in South America, Europe, and North America also reported considerable increase in screen time among children 3–13 years (4).

Parents' behaviors may influence child screen time, and this association has been explained by two theoretical models. One model (15) emphasizes learning through observation, asserting that children develop screen use behaviours through watching their parents and siblings. The second model (16) posits that parents' attitudes and practices creates a microsystem that shapes that of the child. Implementation of routines and limits may lead to establishment of a home environment that discourages unhealthy behaviours in general, which can thereby impact a child's development and relationship with screen use in the broader ecological system.

Various household factors and parental characteristics and practices have been associated with child screen time. For instance, lack of screen-related home rules was associated with exceeding recommended screen time limits in a study of children ages 6–11 years (17). Similar findings were observed among younger children (0–7 years) (18). Meanwhile, a study (*n* = 746; 0–5 years) found parental perception of barriers to limiting child screen time, such as concerns about neighbourhood safety for outdoor play and the demands of busy work days or having multiple children, were associated with higher child screen time (19).

During the COVID-19 pandemic, a cross-sectional study of children (*n* = 1,155) 6–13 years found that a lack of rules regarding screen time was a significant predictor of higher child screen time (20). High levels of parental stress during the pandemic have also been associated with greater child screen time in a Canadian study of children 6–12 years (21). Furthermore, cross-sectional US studies during (22) and prior (23) to the pandemic found associations between higher chaotic household environments and higher child screen time. Higher child screen times were also reported when parents considered screen routines as less or un-important (22).

To our knowledge, there have been no published studies exploring the longitudinal association between routines and limit setting and screen time in children over multiple time points during the COVID-19 pandemic. Given that a paradigm shift towards a more virtual environment is likely to persist beyond the pandemic, this study's findings may help to inform harm-reducing interventions and parenting strategies of screen limits and parental use of routines to limit the potential harms ensued by excessive screen use (10, 24–27).

The primary objective of this study was to examine the longitudinal association between parental use of routines and setting limits with child screen time from May 2020 to May 2021 during the COVID-19 pandemic. The secondary objectives were to examine the association between parental use of routines and setting limits with meeting the Canadian 24-Hour screen time guideline and to describe any change in parental use of routines and setting limits over the study period. We hypothesized that higher parental use of routines and setting limits would be associated with lower screen time and likelihood of meeting the screen time guidelines in children among all age groups included in the study.

## Methods

### Study design and participants

A cohort study using parent-reported repeated measures of independent variables and outcome was conducted in healthy children (0–11 years) through The Applied Research Group for Kids (TARGet Kids!) COVID-19 Study of Children and Families in Toronto, Canada between May 2020 and May 2021. TARGet Kids! is a large practice-based primary care research network in Canada, enrolling healthy children ages 0–5 years from primary health care settings and following them into adolescence (28). At each scheduled well-child visit, parents of participating children are invited to complete an age-specific questionnaire adapted from the Canadian Community Health Survey (CCHS), which includes questions on health and lifestyle factors and child health behaviours such as screen time and physical activity (29). The questionnaires also included socio-demographic information (e.g., family income, employment status, and ethnicity). The TARGet Kids! COVID-19 Study of Children and Families, which launched in April 2020, is nested within the larger TARGet Kids! Cohort. It aims to characterize the COVID-19 pandemic's impact on the children and parents living within the Greater Toronto Area in Canada to inform development of preventative initiatives against COVID-19. Since April 2020, parents participating in the TARGet Kids! longitudinal cohort were invited to complete repeated questionnaires either over the telephone or online via REDCap (30). The questionnaires' contents included physical and mental health and health behaviours of children and parents (e.g., sleep, screen time), parenting practices (e.g., limit setting and routine setting for children), adherence to public health measures, and school and childcare attendance during the COVID-19 pandemic. Informed Verbal consent was provided by all families participating in TARGet Kids!.

### Independent variables

The independent variables of this study were parent-reported general use of routines and limits provided to children, as captured by the bi-weekly-administered question scales, respectively: *throughout the day, I provide my child with a 1 (a clear and orderly routine) to 7 (unstructured free time); I am the kind of parent that 1 (sets limits on what my child is allowed to do) to 7 (lets my child do whatever he or she wants)*.

### Outcomes

The primary outcome variable was parent-reported daily child screen time during the COVID-19 pandemic, captured in the bi-weekly questionnaire. Child screen time was defined as the sum of time spent while: (1) watching TV or digital media (i.e., Netflix, YouTube, web surfing); (2) using social media (i.e., Instagram, Snapchat, Twitter, TikTok); and, (3) playing video games. Screen time for videochatting/face-to-face communication, e-learning or online schoolwork, and watching or reading the news was excluded as these are pro-social activities which may have a positive impact on children during the COVID-19 pandemic. Observations were removed if one of the sub-variables used to compute the overall screen time variable was equal to or exceeded 10 h/day, or if the total screen time was greater than or equal to 12 h/day (31). The secondary outcome variable of meeting vs. not meeting the Canadian 24-Hour screen time guideline (6) was derived from the primary outcome variable, where meeting the guideline was defined as 0 min for <1 years, 60 min or less for 1–5 years, and 120 min or less for >5 years.

### Covariates

Potential confounders identified *a priori* included child age (6), child sex (32–34), maternal ethnicity (32, 35–39), self-reported family income (32, 35–39), unemployment due to the COVID-19 pandemic (32, 35–39), number of siblings (40, 41), number of screen devices in the home (40, 42, 43), parental screen time during the COVID-19 pandemic (40, 44), child pre-COVID-19 screen time (45), and stringent lockdown measures in Ontario.

Parental screen time during the COVID-19 pandemic was obtained from the bi-weekly questionnaire. Implausible parental screen time values (exceeding 24 h/day) were removed and replaced using multiple imputation. This method is based on Fully Conditional Specification, where each variable that is missing data is imputed by a separate model (46). Ever having been unemployed due to the COVID-19 pandemic during this study period was determined from the following question administered on the bi-weekly questionnaire: *Have you been unemployed as a result of the COVID-19 pandemic? (Yes/No).* Stringent lockdown measures were defined as periods when preventative measures were most strict in Ontario and included stay-at-home orders and widespread closures. These periods during the study were May 20, 2020 to Sept 7, 2020 and November 23, 2020 to May 18, 2021. Child age was calculated from date of birth collected at the first administered TARGet Kids! COVID-19 Study of Children and Families questionnaire and the date of questionnaire completion. Child sex, maternal ethnicity, number of siblings, number of screen devices in the home, family income, and child pre-COVID-19 screen time were obtained from the most recently parent-completed questionnaire prior to the COVID-19 pandemic, which ranged from February 2012 to February 2020.

Parenting practices regarding screen time vary by age, with less control typically observed with older children (47–49). Furthermore, the influence of screen time on children also varies by age due to differing developmental abilities and stages (50). Therefore, the current study identified age as a potential effect modifier with strata being defined as <3 years (infants and toddlers), 3–4.99 years (preschoolers), and ≥5 years (school-age children) *a priori* based on the child developmental age groups (51) to allow for better understanding of parental practices and their relationship with child screen time in the context of different stages of development.

### Statistical analysis

Linear and logistic mixed-effects models were fitted using repeated measures of independent variables (parental use of routines and parental use of setting limits) and outcomes (child screen time and meeting the Canadian 24-Hour screen time guidelines). To account for the study's inclusion of some children from the same family, random intercepts for family and subject within family were included. Three models were fitted: (1) unadjusted; (2) adjusted for all covariates but excluding child pre-COVID-19 screen time; and, (3) adjusted for all covariates including child pre-COVID-19 screen time.

Missingness for each covariate was below 15%. Multiple imputation using 15 imputed datasets was performed using the *mice* package in R to account for bias related to missing data (46). Bootstrapping methods with 500 resamples per model were also performed using the *boot* package in R due to heteroscedasticity and non-normality of the model residuals, corresponding non-parametric confidence intervals and empirical *p*-value estimates are reported (52, 53). Statistical significance was set at *p *< 0.05 and all *p*-values were two-tailed. All analyses were conducted using R 4.0.2 (54).

### Ethics approval

This study was approved by the Research Ethics Boards at The Hospital for Sick Children and Unity Health Toronto (TARGet Kids! Cohort Study #10000-12436 and #17-335).

## Results

**Figure 1:**
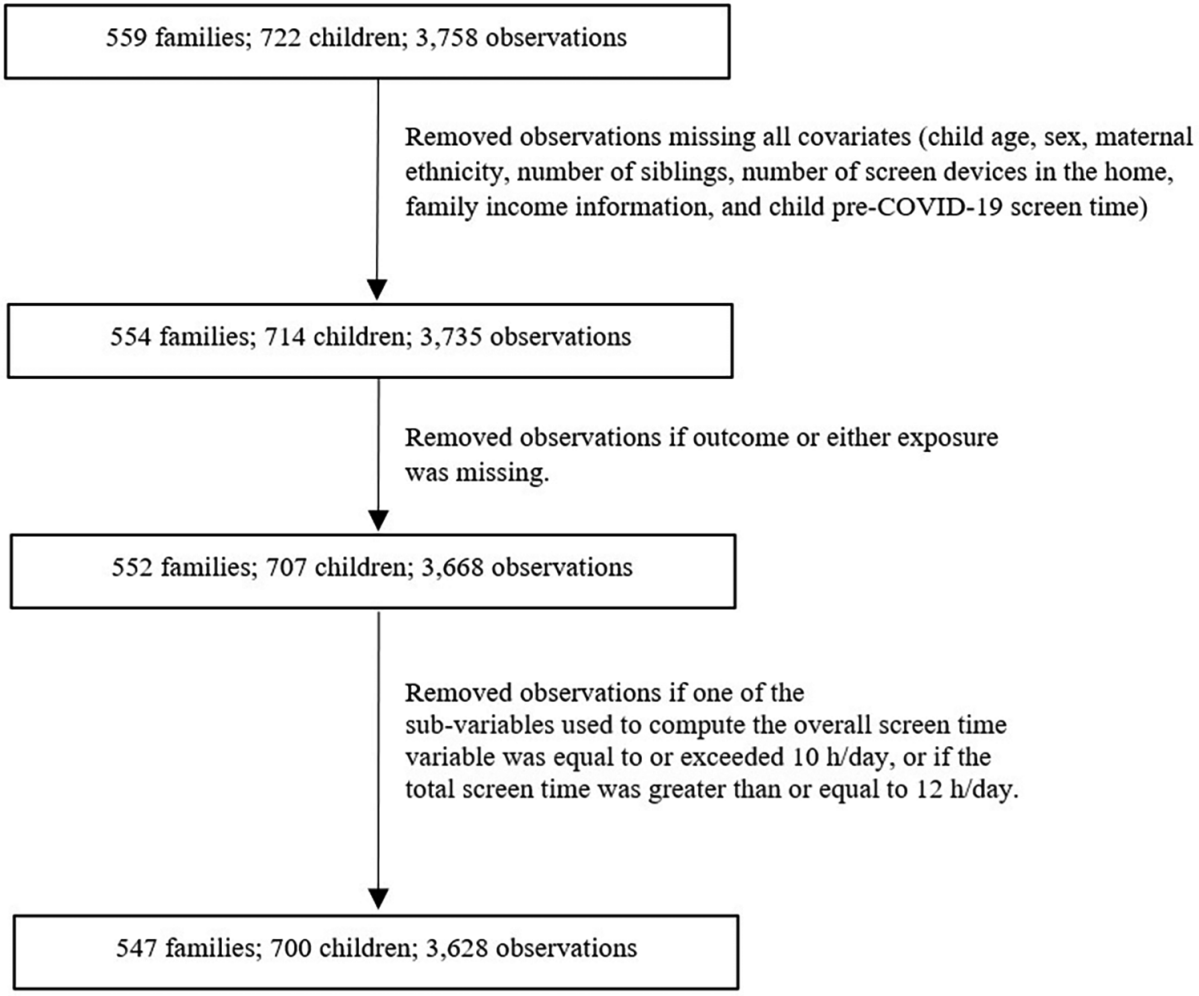
Sample size flow chart.

A total of 700 children with 3,628 observations were included in this study (Figure 1). Descriptive characteristics of the sample are presented in Table 1. Mean age of the children was 5.5 years (SD = 2.7 years), 47.6% of the children were female and 69.4% had mothers who reported European ethnicity.

**Table 1:** Descriptive characteristics of the sample [*N* = 700 (3,628 observations; 547 families)].

Characteristics	Missingness *N* (%)	*N* (%) or mean (*SD*)	
Child age (years)^b^	0 (0.0%)	5.5 (2.7)	
Child sex^a^	0 (0.0%)	Female	333 (47.6)
		Male	367 (52.4)
Maternal ethnicity^a^	102 (14.6%)	European	414 (69.2)
		East Asian	51 (8.5)
		South/Southeast Asian	56 (9.4)
		African	15 (2.5)
		Mixed	38 (6.4)
		Other^f^	24 (4.0)
Self-reported household income^a^	59 (8.4%)	$0 to $39,999	20 (3.1)
		$40,000 to $79,999	76 (11.9)
		$80,000 to $149,999	193 (30.1)
		$150,000+	352 (54.9)
Ever unemployed during COVID-19 (parent completing questionnaire)^c^	27 (3.9%)	Yes	44 (6.5)
		No	629 (93.5)
# of siblings^a^	43 (6.1%)	0	215 (32.7)
		1	341 (51.9)
		2	88 (13.4)
		3	13 (2.0)
# of screen devices at home^a^	0 (0.0%)	7.5 (3.6)	
Level of parental use of routines^c^^,^^d^	0 (0.0)	3.8 (1.5)	
Level of parental use of setting limits^c^^,^^e^	0 (0%)	3.3 (1.5)	
Parental screen time during COVID-19 (hours)^c^	0 (0.0%)	8.4 (4.2)	
Participants meeting screen time guideline^g^	0 (0.0%)	245 (35.0)	
Observations during most stringent lockdown periods^h^	0 (0.0%)	2,965 (81.7)	

^a^ Last measure before COVID-19 (varying between February 7, 2012 and February 13, 2020).

^b^ COVID-19 baseline questionnaire.

^c^ Repeated measures collected during COVID-19 (May 20, 2020–May 18, 2021).

^d^ Question scale: throughout the day, I provide my child with 1 (a clear and orderly routine) to 7 (unstructured free time).

^e^ Question scale: I am the kind of parent that 1 (sets limits on what my child is allowed to do) to 7 (lets my child do whatever he or she wants).

^f^ Other: Arab, Latin American, or Indigenous.

^g^ Canadian 24-Hour screen time guideline: 0 min for <1 years, 60 min or less for 1–5 years, and 120 min or less for >5 years.

^h^ May 20, 2020 to September 7, 2020 and/or November 23, 2020 to May 18, 2021.

**Figure 2:**
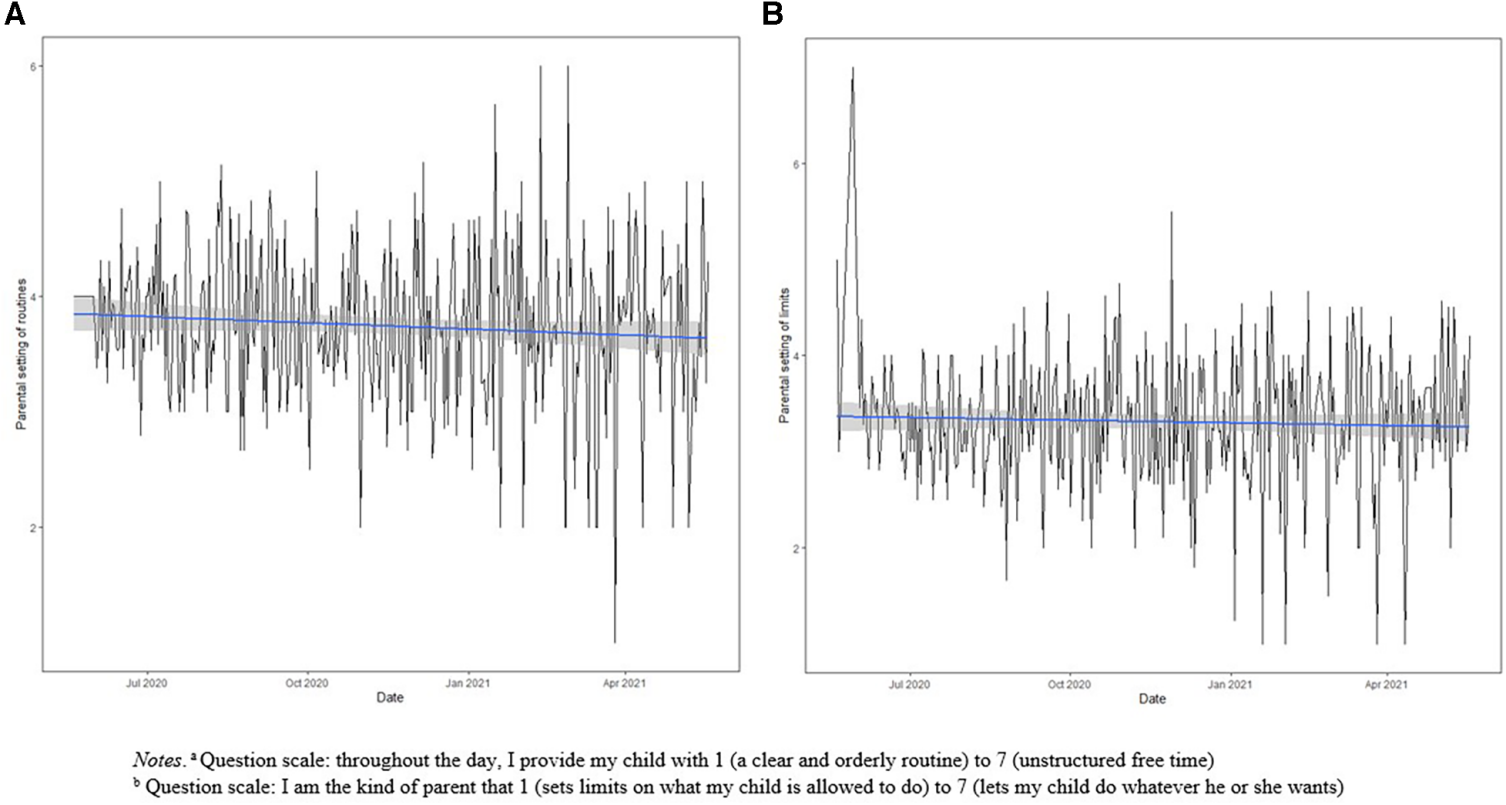
Mean parental use of (**A**) routines and (**B**) limits from May 20, 2020 to May 18, 2021 with linear model and 95% confidence level interval for predictions. (**A**) Question scale: throughout the day, I provide my child with 1 (a clear and orderly routine) to 7 (unstructured free time). (**B**) Question scale: I am the kind of parent that 1 (sets limits on what my child is allowed to do) to 7 (lets my child do whatever they want).

Of the 700 children, 153 (22%) had 1 observation, 101 (14%) had 2 observations, and 446 (64%) had more than 2 observations. The mean number of observations per child was 5 observations. The mean follow-up duration for children with more than 1 observation was 48 days. The mean levels of parental use of routines and setting limits was 3.8 and 3.3 on a 7-point scale, respectively, with higher levels representing less use of routines and setting limits. Overall, the mean change in child screen time, from before to during the pandemic was 51.1 (95% CI: 47.8, 54.9) additional minutes per day. Larger increases were observed among older age groups in a dose-response manner. Mean child screen time during the study period was 126.0 min per day. 52.08% of children <3 years, 25.60% of children 3–4.99 years, and 32.89% of children >5 years met the Canadian 24-Hour screen time guideline on initial and all follow-up questionnaires. The mean level of parental use of routines (*m *< 0.01; *p* = 0.10) and setting limits (*m *< 0.01; *p* = 0.42) remained stable over the study period (Figure 2).

Table 2 presents the associations between parental use of routines, limits, and child screen time, stratified by child age groups. In the ≥5 years age group, for every additional level increase on the Likert scale towards lower parental use of routines there was 5.2 additional minutes of daily child screen time (95% CI: 2.25, 8.73; *p *< 0.01) in the fully adjusted model. In the 3–4.99 years (95% CI: −2.15, 6.28; *p* = 0.36) and <3 years (95% CI: −2.04, 10.39; *p* = 0.16) age groups, there was insufficient evidence of an association between parental use of routines and child screen time. In the ≥5 years age group, for every additional level increase on the Likert scale towards lower parental use of limits, there was 3.3 additional minutes of daily child screen time (95% CI: 0.52, 5.90; *p* = 0.02) in the fully adjusted model. In the 3–4.99 years (95% CI: −3.98, 4.82; *p* = 0.99) and <3 years (95% CI: −3.56, 8.86; *p* = 0.39) age groups, there was insufficient evidence of an association between parental use of setting limits and child screen time.

**Table 2:** Mixed effects models of associations between parental use of routines^a^, limits^b^, and child screen time by developmental age stages (3,628 observations).

	Screen time (min per day) during COVID-19								
	Unadjusted			Adjusted for covariates^c^ − pre-COVID-19 screen time			Adjusted for covariates^c^ + pre-COVID-19 screen time		
	*B*	95% CI	*p*-value	*B*	95% CI	*p*-value	*B*	95% CI	*p*-value
Routines									
<3 years (591 observations)	3.37	−3.52,9.17	0.36	5.08	−1.80, 10.84	0.13	4.61	−2.04, 10.39	0.16
3–4.99 years (907 observations)	2.65	−1.44, 7.28	0.26	2.29	−1.79, 6.74	0.32	2.10	−2.15, 6.28	0.36
≥5 years (2,130 observations)	4.84	1.50, 8.28	<0.01	5.25	2.24, 8.73	<0.01	5.25	2.25, 8.73	<0.01
Limits									
<3 years (591 observations)	3.26	−3.72, 8.65	0.41	3.77	−3.37, 9.34	0.32	3.41	−3.56, 8.86	0.39
3–4.99 years (907 observations)	0.88	−3.27, 5.82	0.77	0.34	−3.71, 5.28	0.90	0.03	−3.98, 4.82	0.99
≥5 years (2,130 observations)	2.70	−0.10, 5.34	0.05	2.98	0.09, 5.58	0.03	3.30	0.52, 5.90	0.02

^a^ Question scale: throughout the day, I provide my child with 1 (a clear and orderly routine) to 7 (unstructured free time).

^b^ Question scale: I am the kind of parent that 1 (sets limits on what my child is allowed to do) to 7 (lets my child do whatever he or she wants).

^c^ Covariates: child sex, maternal ethnicity, most recently reported pre-COVID-19 family income, employment status during COVID-19 (repeated measures), number of siblings, number of screen devices at home, parental screen time during COVID-19 (repeated measures), stringent lockdown measures; results were stratified by age (<3, 3–4.99, ≥5 years) as defined *a priori* based on the child developmental age groups.

Table 3 presents the associations between parental use of routines, setting limits, and meeting the Canadian 24-Hour screen time guideline. In the fully adjusted model, among children <3 years and ≥5 years, for every additional level increase on the Likert scale towards lower parental use routines, the odds of meeting the screen time guideline was lower in children <3 years and ≥5 years (OR = 0.59; 95% CI: 0.38, 0.88; *p* = 0.01 in children <3 years; OR = 0.76; 95% CI: 0.67, 0.87; *p *< 0.01 in children ≥5 years). There was insufficient evidence of an association among children 3–4.99 years (OR = 0.96; 95% CI: 0.77, 0.1.23; *p* = 0.75). The odds of meeting the screen time guideline was only lower in children ≥5 years for every additional level increase on the Likert scale towards lower parental use of limits (OR = 0.86; 95% CI: 0.78, 0.94; *p *< 0.01). There was insufficient evidence of an association among children <3 years and 3–4.99 years (OR = 0.65; 95% CI: 0.39, 1.01; *p* = 0.07). and 3–4.99 years (OR = 1.10; CI: 0.89, 1.35; *p* = 0.38).

**Table 3:** Mixed effects models of associations between parental use of routines^a^, limits^b^, and meeting the Canadian 24-hour screen time guideline^c^ (3,628 observations).

	Meeting the Canadian 24-Hour screen time guideline during COVID-19								
	Unadjusted			Adjusted for covariates^d^ − pre-COVID-19 screen time			Adjusted for covariates^d^ + pre-COVID-19 screen time		
	OR	95% CI	*p*-value	OR	95% CI	*p*-value	OR	95% CI	*p*-value
Routines									
<3 years (591 observations)	0.62	0.43, 0.89	0.01	0.55	0.35, 0.84	<0.01	0.59	0.38, 0.88	0.03
3–4.99 years (907 observations)	0.91	0.73, 1.10	0.35	0.94	0.75, 1.19	0.61	0.96	0.77, 1.23	0.75
≥5 years (2,130 observations)	0.79	0.68, 0.92	<0.01	0.75	0.65, 0.85	<0.01	0.76	0.67, 0.87	<0.01
Limits									
<3 years (591 observations)	0.64	0.43, 0.98	0.04	0.62	0.38, 0.96	0.04	0.65	0.39, 1.01	0.07
3–4.99 years (907 observations)	1.01	0.82, 1.24	0.93	1.06	0.86, 1.29	0.53	1.10	0.89, 1.35	0.38
≥5 years (2,130 observations)	0.92	0.83, 1.01	0.10	0.86	0.77, 0.94	<0.01	0.86	0.78, 0.94	<0.01

^a^ Question scale: throughout the day, I provide my child with 1 (a clear and orderly routine) to 7 (unstructured free time).

^b^ Question scale: I am the kind of parent that 1 (sets limits on what my child is allowed to do) to 7 (lets my child do whatever he or she wants).

^c^ Canadian 24-Hour screen time guideline: 0 min for <1 years, 60 min or less for 1–5 years, and 120 min or less for >5 years.

^d^ Covariates: child sex, maternal ethnicity, most recently reported pre-COVID-19 family income, employment status during COVID-19 (repeated measures), number of siblings, number of screen devices at home, parental screen time during COVID-19 (repeated measures), stringent lockdown measures; results were stratified by age (<3, 3–4.99, ≥5 years) as defined *a priori* based on the child developmental age groups.

## Discussion

In this longitudinal study of a community sample of Canadian children under 12 years of age, we examined the relationships between parental use of routines and setting limits with child screen time during the COVID-19 pandemic from May 2020 to May 2021. Mean child screen time during the pandemic increased compared to pre-pandemic for all age groups (<3, 3–4.99, ≥5 years) and larger increases were observed with each older age group. Our results provided evidence that lower parental use of routines and setting limits was associated with higher child screen time and lower odds of meeting the Canadian 24-Hour screen time guideline among the ≥5 years age group before and after adjusting for pre-COVID-19 child screen time in addition to other covariates. The magnitudes of screen time increase for each single additional level increase on the Likert scale towards lower parental use of routines and limits, though small, demonstrate a more clinically relevant increase when comparing the degree of routine and limit implementation at opposite ends of the spectrum.

Restrictions implemented to curb the spread of COVID-19 have resulted in increased demands of homeschooling or supervising children while adapting to work-related changes. This has put considerable strain on parents, as reflected by reports of poorer mental health by parents (55, 56). As such, ensuring that children meet recommended screen time guidelines (6) may be especially challenging. Specifically in Ontario, stay-at-home measures and some of the longest school and daycare closures implemented throughout periods in 2020–2021 among the Canadian provinces must be considered as factors contributing to increases in child screen time. Beyond the pandemic and related restrictions, it is important to also acknowledge that many families will continue to face barriers that make meeting the screen time guideline for children very difficult, such as lack of affordable alternate activities and parental fatigue and stress (57). The findings of our study can thus contribute to informing potential strategies that help parents implement methods that encourage healthy screen time when possible as there is likely to be a fine balance between obtaining the benefits of screen use such as for prosocial activities and learning (58), and its detrimental effects such as sedentary behaviour and mental health symptoms, particularly during the COVID-19 pandemic (10). As the transition from pandemic to post-pandemic circumstances occurs, these strategies may continue to be relevant as actionable ways of encouraging healthy screen time. In addition, mitigation strategies to reduce screen time should not fall entirely on caregivers; public health strategies to reduce infection rates during and following the pandemic need to consider unintended consequences of measures such as stay-at-home orders, physical isolation protocols, and distance-based online education on children (59).

When considered through the lens of the Bronfenbrenner's ecological systems theory (16), establishment of routines and limits contribute to the home environment and may also represent parents' attitudes towards screen use. These parental practices can therefore impact a child's relationship with screen use in the home environment and the broader environment beyond it. Our findings also align with those of previous studies that have found increases in child screen time from prior to during the COVID-19 pandemic (2–4). They also build upon those of previous studies that have examined the relationship between household chaos and child screen time (22, 23), where household chaos is characterized by factors such as high levels of background stimulation, lack of routine or structure in daily activities, and fast-paced family life (60, 61). In contrast to the study by Emond et al. (2018), which employed the Confusion, Hubbub and Order Scale to characterize household chaos, we did not observe sufficient evidence supporting a relationship between parental use of routines and child screen time in children <5 years. This may be due to insufficient power in our study and may also highlight the roles that other household chaos elements. However, our findings partially align with those of Kracht et al. (2021) for children ≥5 years, which observed an association between more household chaos and greater child screen time.

Our study's findings are also consistent with studies conducted prior to and during the COVID-19 pandemic that have observed greater screen time with less screen-related rules in children ≥5 years (17, 18, 20). However, our study found insufficient evidence of an association between parental use of setting limits and child screen time among children <5 years. This finding may be related to our stratification of analyses by the child developmental age groups, which provides granularity related to the varying abilities of the different developmental stages (50), at the expense of statistical power. It may also be partially explained by varying parental interpretations of what it means to set limits vs. screen-related rules for children at different developmental stages. For a child <5 years, “setting rules related to screens” and “setting limits” may be interpreted as very similar concepts. In contrast, parents may not set limits for their children <5 years because this age group is not yet highly autonomous nor independent, but they may have screen-related parenting rules that they follow to ensure their child is not exposed to screen time (51).

Notably, the estimated association between parental use of setting limits and child screen time was similar to that between parental use of routines and child screen time for children ≥5 years. This may be explained by correlation between routine and limit establishment and how they directly or indirectly relate to screen time. For instance, while limits are typically set for activities considered unhealthy and therefore often explicitly include screen time directly (62–65), routines regarding bedtime and physical activity are not directly related to but also appear to play a role in determining screen time among children (66).

The proportion of children meeting the Canadian 24-Hour screen time guideline varied by age group and was larger than that observed in other Canadian studies during the COVID-19 pandemic, though these studies focused on children and adolescents >5 years (67, 68). Compared to studies prior to the pandemic, similar or smaller proportions of participants in our study met the screen time guideline (6, 69, 70). Assessing the associations between parental routine and limit setting with meeting the guideline provides clinical relevance to the quantified changes in amount of screen time as it has been established that meeting the guideline confers a number of health benefits (3, 7, 8). Our findings suggest that parental use of routine and limit setting may play a positive role in helping children meet the screen time guideline, particularly among the older age groups of 3–4.99 years and ≥5 years.

This study is one of the first to evaluate the longitudinal link between parental use of routines and limits with child screen time during the COVID-19 pandemic. Strengths of this study include use of a cohort design, repeated measure to improve estimates of associations, and adjustment for multiple confounders including child screen time prior to the COVID-19 pandemic. Limitations include inability to make causal links between parental use of routines and setting limits with child screen time given its observational design and the potential of unmeasured confounders. Measures of the independent variables and outcomes were self-reported by parents and may have therefore been subject to self-reporting bias that could reduce the validity of the results. Furthermore, the measures represent parental perceptions and are also subject to interpretation. As such, they may not correlate with objective measurement of the variables. Finally, this study is embedded in a primary care research network that mainly constitutes children of European ethnicity residing in an urban center and belonging to higher socioeconomic classes. Therefore, replications studies in different contexts are needed as the findings of this study may not be generalizable to other populations, such as lower income and rural or suburban populations.

## Data Availability

The raw data supporting the conclusions of this article will be made available by the authors, without undue reservation.
